# HIV/AIDS mitigation strategies and the State in sub-Saharan Africa – the missing link?

**DOI:** 10.1186/1744-8603-2-1

**Published:** 2006-01-17

**Authors:** Abdu Mohiddin, Deborah Johnston

**Affiliations:** 1 Division of Health and Social Care Research, Guy's, King's and St Thomas' School of Medicine, Kings College London, London SE1 3QD, UK; 2 Department of Economics, School of Oriental & African Studies, London WC1H 0XG, UK

## Abstract

**Background:**

The HIV/AIDS pandemic in sub-Saharan Africa is widely recognised as a development disaster threatening poverty reduction, economic growth and not merely a health issue. Its mitigation includes the societal-wide adoption and implementation of specific health technologies, many of which depend on functional institutions and State.

**Discussion:**

Donor and International Institutions' strategies to mitigate HIV/AIDS in sub-Saharan Africa are premised on a single optimal model of the State, one which focuses on the decentralised delivery of public goods alone (such as healthcare) – the service delivery state. The empirical evidence, though sparse, of "successful" and "unsuccessful" sub-Saharan Africa states' performance in mitigating HIV/AIDS does not support this model. Rather, the evidence suggests an alternative model that takes a country context specific approach – encompassing political power, institutional structures and the level of health technology needed. This model draws on the historical experience of East Asian countries' rapid development.

**Summary:**

For international public health policies to be effective, they must consider a country tailored approach, one that advocates a coordinated strategy designed and led by the State with involvement of wider society specific to each country's particular history, culture, and level of development.

## Background

The HIV/AIDS epidemic in sub-Saharan Africa (SSA) is a human and development disaster [1]. Now significant donor resources are available to fund mitigation strategies [2-4]. However, the approach to HIV/AIDS in SSA has been criticised as being based on health policies from industrialised countries which treat HIV/AIDS differently from other sexually transmitted infections – HIV exceptionalism. Some have called for a new strategy based on a public health model that rejects uniform approaches to the epidemic, emphasises social justice and public health, rather than only individual human rights [5]. We ask, are there limitations too, in the model of the State that current mitigation policies assume and insist are optimal for SSA? Though the evidence-base is sparse, our findings suggest that persisting with the current model risks possible failure of the donor mitigation strategies – there is an imperative to consider countries' diversity and context in designing mitigation strategies.

Donors are funding the implementation and expansion of anti-HIV/AIDS programmes incorporating treatment for HIV/AIDS (antiretroviral therapy or ARV). They are generally targeted at low-income SSA countries that have a high HIV/AIDS prevalence and have conditions attached that insist on adopting the current state model – the Service Delivery State (SDS).

We begin by outlining the necessary technological elements of an effective anti-HIV/AIDS strategy, then describe the SDS, and review the empirical evidence of SSA State's performance in HIV/AIDS mitigation. Finally we explore a different State model and the consequences of persisting with the international mitigation strategies.

### Mitigation strategies

Mitigation strategies exist to control, treat, and prevent HIV/AIDS and are a form of technology (including institutional organisation) that a State needs to adopt in order to achieve mitigation [6]. Mitigation encompasses more than health (e.g. agricultural, industrial interventions) but here we focus on health. This technology varies in complexity and, to simplify, includes prevention, treatment for sexually transmitted infections (STI's), and HIV/AIDS therapy (table 1). Ideally, a comprehensive strategy would have all three technologies. Prevention aims to educate and change individual and group behaviours. The treatment of sexually transmitted infections (STI's) also reduces the risk of HIV transmission. More technical, however, is ARV, which involves taking a regimen of drugs daily, adhering to this schedule, and the treatment and monitoring of opportunistic infections. ARV is effective (and lifelong), making HIV/AIDS a chronically managed disease.

**Table 1:** Health technology for mitigating HIV/AIDS (simplified to illustrate)

	**Health technology interventions/mitigation strategies**		
	**Behavioural and Educational prevention**	**Treatment of Sexually Transmitted Infections**	**Antiretroviral Therapy**

**Human capital/expertise**	Field-based (including peer) health educators and promoters	Nursing-level	Medical-level
**Physical Infrastructure**	Minimal, community meeting, radio/TV campaigns	Health centres	Health centres, hospital and laboratory facilities
**Organisation**	Local community level	Intermediate – drug and monitoring network	Coordinated drug and laboratory network, treatment monitoring
**Technology, resource, and coordination needs**	Low	Medium	high

Source: Authors

The State plays an important role in the interventions described above that other providers (say, non-governmental organisations (NGO's) or private) either cannot or have a limited role. Many of these technologies are dependent on a functioning State and institutional structure that reflects these technological requirements. The State is the legitimate body that can lead societal-wide efforts to prioritise and co-ordinate anti-HIV/AIDS activities. These would include providing leadership, legislation, and enforcement regarding sensitive gender, cultural and sexual practices and roles. The State has responsibility for the health of its citizens and its function also encompasses negotiation of intellectual property rights on say, drug patents, including the use of emergency rights available in the World Trade Organisation rules, important not just for current health technologies, but future innovations too [7].

The model of the State that the mitigation strategies assume is the SDS which views the State's role as providing public goods (health, education, physical infrastructure, regulation) with the market delivering all other goods and services [8]. Allied to this are democratic accountability, institutional decentralisation (defined as the devolution of decision-making to sub-units i.e. closer to the ground, thus able to address needs and deliver services more equitably and effectively), and good governance reforms (which include anticorruption measures – like judicial independence), with civil society participation to provide a voice and discipline the State (table 2). The SDS assumes that the highest level of technology is achievable through decentralised institutions (in this case ARV).

**Table 2:** summarising the Service Delivery State

	**Service Delivery State**
Political arrangement	Democracy (multi-party), good governance
Institutional structure	Decentralised for service delivery
Technology achievable	Highest level – antiretroviral therapy

Source: Authors

### The evidence of State performance in mitigating HIV/AIDS in SSA

The experience of mitigation in SSA can be divided into those countries that have seen some "success" namely an active and sustained response that has stalled (and possibly reduced) the prevalence of HIV/AIDS and those that have not achieved this. We reviewed the published literature and attempt to compare these responses with the SDS model and have focused on low-income SSA countries as they bear the bulk of the disease burden and are the target of the mitigation strategies.

#### The "successes"

The "successful" countries are generally acknowledged as Uganda and Senegal. The evidence [9-12] points to the following successful features: strong political leadership from the president and others in government; political stability, a coordinated and agreed nationally "owned" strategy involving non-State (especially faith based) actors over time; the distribution and disposition of political power in society (the political settlement) is committed to a strategy which is in turn context specific (rather than driven by internationally determined policies too focused on technical solutions); an institutional structure matched to the political settlement; and, investment over time in the health infrastructure to produce a step-wise adoption of health technology recognising the capacity limitations present in a country (e.g. initial centralised provision).

#### The "failures"

In contrast the "unsuccessful" countries, those that have not mounted a meaningful mitigation response reviewed here are Zambia, Namibia, and Ethiopia. The evidence [13-15] finds the following.

A failure of political will to tackle influential groups that may block mitigation (e.g. traditional rulers, religious groups), and, build a committed response to the epidemic. Difficulties with decentralisation include a lack of supervision and control, little interest (and hence funds) in HIV/AIDS by some local decision-makers, inequities in service provision, a need for improved information sharing between national structures and the decentralised entities. The evidence demonstrates the existence of a lack of clarity regarding the powers and functions of decentralised levels of government, poor financial frameworks for fiscal decentralisation, and poor capacity of local officials and councillors at a local level. Further, national mandates were not well clarified to local levels with service delivery assigned to specific government agencies (e.g. prevention of maternal-to-child HIV transmission, nutrition). Building community coordination and integration activities are lengthy and intensive yet were largely unfunded. However, some promising developments of decentralisation were noted – integrated local level planning, and local HIV/AIDS coordination systems linking NGOs with local government to produce a more coherent response.

#### Insights from SSA Political Economy and mitigation policies beyond SSA

Parkhurst reviews the experiences of several countries in tackling HIV/AIDS and emphasises that to understand a country's response to the epidemic one must look to the context, namely the national culture, political environment and actors involved in implementing policy [16,17]. Parkhurst critiques the prevailing international policy guidance as based on a policy model that sees response to disease as determined by health need, and implementation as a local technical function; instead history shows how HIV/AIDS is no different from other issues in the necessity of understanding local contexts to produce effective policies.

Van de Walle in a major survey studied African political economy in the context of donor imposed institutional reforms of the past two decades [18]. He finds that less donor-supported institutional reform had happened than expected, instead – paradoxically – elites have been strengthened by such reforms. Reform periods have been characterised by change and uncertainty raising the chance of corruption. The net effect has been a decline in State capacity coupled with weaker accountability and transparency.

These findings are echoed by Szeftel who sees institutional reforms as imposing rules and regulations developed for rich liberal democracies on very different environments like SSA [19]. These reforms are concerned with stability (of markets, private enterprise and civil society) and change ultimately fails as the existing interests are left intact.

## Discussion of the findings (table 3)

**Table 3:** summarising the empirical evidence of low-income SSA States' success in mitigating HIV/AIDS

	**"Successful" States**	**"Unsuccessful" States**
Political arrangement	Varied: democracy (Senegal, multiparty), authoritarian (Uganda)	Varied: democracy (multiparty, Zambia), others including authoritarian (Namibia, Ethiopia)
Institutional structures	Centralised (now decentralising)	Varied (decentralised and centralised)
Technology achieved	STIs, some ARV through centralised institution	Some behaviour/education, centralised STIs, sparse centralised ARVs

Source: Authors

STI – Treatment of sexually transmitted infections

ARVs – antiretroviral treatment

Uganda and Senegal achieved more success as they had a clear goal to tackling HIV and had commitment from important societal stakeholders that the State led and encouraged. Both countries recognised the limitations of their capacity and adopted health technologies appropriately (and attempted a sequenced development over time). Both developed a mitigation strategy suitable for their context and were able to some extent to draw donors into this vision. The other countries, in contrast, did not have such a clear and wide political commitment; rather there was a superficial articulation of this, if at all. Ethiopia, for example, suffered from political instability and mitigation at the lowest technological level (behavioural/educational interventions) was barely delivered.

The evidence emphasises the diversity of political arrangements in both the successful and unsuccessful groups, but with success due to a committed pro-mitigation political settlement. Institutional decentralisation (as in Namibia) is to be carefully considered as the findings point to gaps in capacity (also accountability and coordination) undermining mitigation. The experience of Senegal, for example, points to the benefits of centralisation as a way of delivering higher technology interventions in a capacity constrained environment.

Importantly the findings emphasise the primacy of political commitment and that it need not be democratically based. There is no clear evidence of the benefit of democracy and decentralisation in delivering mitigation (including ARV use). All this points to the inadequacy of the SDS suggesting that a different model of the State may be necessary to understand the context in a particular country and then develop a successful strategy. This alternative model has to address specific issues in State failure. Unfortunately, there are few studies of country performance in tackling HIV/AIDS particularly from a political economy perspective and the ones that are available (and reviewed here) tend to be reports which may not have been subject to peer-review.

However the experience of Uganda and Senegal demonstrates that their approach to HIV mitigation (as a development challenge beyond merely health) has similarities with the East Asian States' significant successes in their social and economic development. Political economic studies of this success have highlighted a possible alternative state model – the Developmental State (DevS).

### Issues and determinants of State failure

The DevS sees the critical area of state failure as the lack of adequate institutional and political capacity to produce a dynamic societal transformation towards greater social and economic development. Such states are described as having two elements- ideological and structural [20]. The ideological drive is a commitment to development by the State with other key societal actors being willing participants (i.e. the political settlement is committed to development; in the HIV context, a commitment to mitigation as seen in Uganda and Senegal). The structural component refers to the capacity of the state to implement policies effectively, with this capacity being determined by other constituent capacities – institutional, technical and political.

The political settlement is key, successful States are a task of "political engineering" as much as "institutional engineering" [21]. The mismatch between the political settlement and institutions can explain State failure when institutions from one context are placed in another (as in the SDS approach using an industrialised country health policy solution template [5]). Effective institutional enforcement requires institutional capacity and compatibility with the underlying political settlement. These descriptions have a resonance with the earlier findings on successful and unsuccessful mitigation experiences.

The SDS recognises this political importance in a limited way, hence the good governance institutional agenda. But, there is a distinction to be made between power and institutional structures. The distribution of power may not match institutional "paper" structures. The establishment of formal institutions (say, a National AIDS Control Committee) may be unsuccessful in this context where the informal institution of power is with competing interest groups. Spending on mitigation may be diverted or contested by these groups, and there is also the possibility of the fragmented and unproductive acquisition of health technology rendering it less effective in a few years e.g. through ARV resistance.

Different institutional arrangements of a State lead to different consequences for what the State can achieve or not [21]. In making the centralise/decentralise decision, the following should be considered – the type of technology (and wider social benefits and co-ordination problems), the level of overall development of a country which would indicate the capacity of the State and the nature of the bureaucracy. For example, an eroded bureaucracy (e.g. due to the HIV epidemic) makes implementation of institutional reforms challenging.

Finally history has shown that SSA State elites have tended to be strengthened following imposed institutional reforms. Particular groups may gain preferential access to ARV's thus entrenching inequalities perhaps preventing any new social contract arising from the epidemic. Donors insist on decentralisation, and in a capacity-constrained environment this means reliance on NGOs and non-State actors risking a lack of accountability, poor regulation/performance monitoring, a variation in standards, inequities in access and uncertainty over funding timescales

## Summary

This is a broad look at the State and mitigation, limited by the evidence-base on SSA State performance. Whilst more research is indicated, little support for the current SDS model is found. Rather the DevS model is preferred as it recognises that a context specific approach is necessary, taking into account the existing political settlement and institutional and technological solutions, along with a long timescale coordinated strategy designed and led by the State with involvement of wider society specific to its particular history and culture. International institutions and donors should reconsider promoting a "one-size-fits-all" approach to mitigation; especially overlooking the crucial role of the State.

A rethink considering the political economy of the State would lead to more effective and sustainable HIV/AIDS mitigation strategies in SSA countries. For instance, States can act to introduce a new form of health financing/taxation, acquire trade and debt concessions, invest in human capital and local pharmaceutical industries and so on. The battle against HIV/AIDS in SSA States is no less than a social transformation and as such should be linked to wider development goals to be truly effective.

## Competing interests

The author(s) declare that they have no competing interests.
